# The Modulatory Effects of Transcranial Alternating Current Stimulation on Brain Oscillatory Patterns in the Beta Band in Healthy Older Adults

**DOI:** 10.3390/brainsci14121284

**Published:** 2024-12-20

**Authors:** Kenya Morales Fajardo, Xuanteng Yan, George Lungoci, Monserrat Casado Sánchez, Georgios D. Mitsis, Marie-Hélène Boudrias

**Affiliations:** 1 School of Physical and Occupational Therapy, McGill University, Montréal, QC H3G 1Y5, Canada; kenya.moralesfajardo@mail.mcgill.ca; 2 Center for Interdisciplinary Research in Rehabilitation of Greater Montreal (CRIR), Montréal, QC H3S 1M9, Canada; xuanteng.yan@mail.mcgill.ca (X.Y.); george.lungoci@mail.mcgill.ca (G.L.); monserrat.casado@mail.mcgill.ca (M.C.S.); 3 Department of Bioengineering, McGill University, Montréal, QC H3A 0E9, Canada; georgios.mitsis@mcgill.ca; 4 Integrated Program in Neuroscience, McGill University, Montréal, QC H3A 1A1, Canada

**Keywords:** tACS, aging, MRBD, EEG, movement, beta oscillations, non-invasive brain stimulation

## Abstract

**Background:** In the last few years, transcranial alternating current stimulation (tACS) has attracted attention as a promising approach to interact with ongoing oscillatory cortical activity and, consequently, to enhance cognitive and motor processes. While tACS findings are limited by high variability in young adults’ responses, its effects on brain oscillations in older adults remain largely unexplored. In fact, the modulatory effects of tACS on cortical oscillations in healthy aging participants have not yet been investigated extensively, particularly during movement. This study aimed to examine the after-effects of 20 Hz and 70 Hz High-Definition tACS on beta oscillations both during rest and movement. **Methods:** We recorded resting state EEG signals and during a handgrip task in 15 healthy older participants. We applied 10 min of 20 Hz HD-tACS, 70 Hz HD-tACS or Sham stimulation for 10 min. We extracted resting-state beta power and movement-related beta desynchronization (MRBD) values to compare between stimulation frequencies and across time. **Results:** We found that 20 Hz HD-tACS induced a significant reduction in beta power for electrodes C3 and CP3, while 70 Hz did not have any significant effects. With regards to MRBD, 20 Hz HD-tACS led to more negative values, while 70 Hz HD-tACS resulted in more positive ones for electrodes C3 and FC3. **Conclusions:** These findings suggest that HD-tACS can modulate beta brain oscillations with frequency specificity. They also highlight the focal impact of HD-tACS, which elicits effects on the cortical region situated directly beneath the stimulation electrode.

## 1. Introduction

Advanced age often comes with a decline in sensorimotor control and functioning that affects the ability to perform activities of daily living. Indeed, it has been shown that movements become slower and/or less accurate and more cognition-dependent as we age [1]. Motor declines overall significantly impact motor independence, which is essential for older adults’ quality of life and interactions with their environment. Additionally, behavioral evidence indicates that aging is frequently linked to slower movements, reduced capacity for learning new motor skills, and diminished ability to adjust a movement plan after initiation [2]. In fact, Mild Parkinsonian Symptoms (**MPSs**), such as rigidity, bradykinesia, and tremor, are commonly diagnosed during clinical examination of older adults who do not have a diagnosed neurological disease [3].

Advances in neuroimaging techniques have made contributions to a better understanding of the aging brain. For instance, aging impacts brain structure, leading to a decrease in gray and white matter volume, along with an increase in cerebrospinal fluid in ventricles, fissures, and sulci [4,5]. These aging-related processes affect almost the entire cortex and underlying white matter, with a steeper decline in the primary motor cortex (**M1**) and frontal subcortical white matter [6]. For instance, aging is also associated with a complex pattern of atrophy [7], demyelination [8], free tissue water, and iron reduction within somatosensory and motor areas [6]. Moreover, age-related atrophy of motor cortical regions and the corpus callosum has been shown to coincide with motor declines such as balance gait deficits and coordination deficits [9].

Sensorimotor cortex oscillations measured by electroencephalography (**EEG**) in the beta band (13–30 Hz) are a predominant feature of movement production and have been shown to be generated by local field potentials within the motor cortex [10]. Beta oscillations exhibit a robust pattern of movement-related changes, such as pre-movement beta Event-Related Desynchronization (**ERD**), Movement-Related Beta Desynchronization (**MRBD**), and post-movement beta Event-Related Synchronization (**ERS**) or beta rebound [11,12]. In terms of how these oscillations relate to motor performance, an association between MRBD and the accuracy with which subjects performed a bimanual task has been demonstrated, where subjects with more negative MRBD values exhibited worse task performance [13]. Greater MRBDs were also shown to correlate with a longer movement duration to complete a finger-tapping sequence [14]. In older adults, a greater (i.e. more negative) MRBD in both motor and premotor areas has been observed in subjects performing cued finger button presses [15,16] and handgrip tasks [13]. Additionally, older age has been associated with greater baseline beta power (15–29 Hz) at rest [17], suggesting that the alterations in brain structure and biochemistry during aging could be the reason behind the observed altered neural activation patterns. Given the association between movement production and beta band features, there is a high interest in modulating these oscillations non-invasively to improve motor ability and performance in older adults.

Transcranial alternating current stimulation (**tACS**) is a non-invasive brain stimulation (**NIBS**) technique that can alter oscillatory brain rhythms through synchronization of neural networks in a frequency-dependent manner [18]. This method is believed to entrain endogenous brain oscillations through the synchronization of two oscillatory systems that occurs when a driving external oscillatory force coordinates with another oscillating system [19,20,21,22]. The effects of tACS depend on key parameters: stimulation location, intensity, and frequency [23]. In terms of location, the acquisition of motor skills is linked to a number of cortical and subcortical brain regions, but among these, M1 is thought to play a central role [24,25,26,27], making it a popular target for neurostimulation. Regarding intensity, it is generally set between 1 mA and 2 mA because it is well tolerated and it has been shown to modulate cortex excitability and alter cognitive function [28]. Additionally, using higher intensities raise concerns about safety and side effects [29]. Regarding frequency, motor cortex activity during movement predominantly oscillates at 20 Hz (beta band) [30] and 70 Hz (gamma band) [31]. Beta band activity within the motor system has been linked to an antikinetic role, as it is associated with slower voluntary movements in both healthy individuals [21,32,33] and those with motor disorders [34]. A reason for this may be that, in the cortex–basal ganglia circuit, beta activity is associated with promoting tonic rather than voluntary movement [35,36]. Also, motor impairments in Parkinson’s disease (**PD**) have also been linked to elevated beta band activity in the motor cortex and subthalamic nucleus [37]. In contrast, gamma band activity is thought to be prokinetic, as it increases in the basal ganglia–cortical motor circuit during voluntary movement [38]. Behaviorally, the use of 20 Hz tACS has been shown to slow voluntary movement, while 70 Hz tACS enhances motor learning along with an increase in beta power [32]. However, these results involved participants in the younger range (32.7 ± 6.8 years) and the influence of these tACS frequencies on aging-related brain neural activity has not yet been studied.

Other NIBS techniques, such as transcranial direct current stimulation (**tDCS**), have been shown to have greater effects on motor performance when applied during a motor task, a technique often called *online stimulation*, compared to before the motor task [39]. There is also evidence that applying tDCS during practice triggers effects that outlast the stimulation period and facilitate neuroplasticity [28]. Previous studies have reported mixed results regarding the effects of NIBS on young adults, and these results cannot be easily transferred to older adults. The stimulation sites and frequencies that modulate brain oscillatory activity in young adults may not result in the same effect in older adults, and functional reorganization of the aging brain may be an explanation [40].

In recent years, standard double-electrode tACS has shown limitations in controlling the stimulation focus and intensity. The use of different electrode montages, such as High-Definition tACS (**HD-tACS**), has allowed more precise stimulation control. Prior research has indicated that HD-tACS yields a more pronounced focalization of its effects through multiple smaller electrodes, possibly resulting from reduced distribution of the electrical field compared to conventional tACS [41,42]. Notably, online HD-tACS, applied during a motor task, induces phase- and frequency-dependent effects on cortical excitability [43,44].

After-effects on brain oscillations are a common outcome following tACS [45]. For instance, 10 Hz tACS stimulation of the parieto-occipital area resulted in an enhancement of the EEG-recorded alpha amplitude during the stimulation and this effect was seen to last at least 30 min after a 10 min stimulation period [46,47]. Other NIBS techniques, such as tDCS, have induced long-lasting excitability elevations in the human motor cortex [48], and in animals, a stimulation period of 5 to 30 min causes an effect lasting for hours after the end of stimulation [49].

This study aims to explore the after-effects of 70 Hz and 20 Hz HD-tACS on beta brain oscillatory patterns in healthy older adults. Based on the previously mentioned effect of tACS on motor performance on younger cohorts and how their beta brain oscillations differ from older ones, we hypothesized that 70 Hz HD-tACS would decrease resting-state beta power and promote a more positive MRBD (lower desynchronization). Conversely, we hypothesized that 20 Hz HD-tACS would increase beta power at rest and induce more negative MRBD (higher desynchronization).

## 2. Materials and Methods

### 2.1. Participants

In this single-blinded, sham-controlled study, 15 healthy individuals (7 males and 8 females) over 65 years old (age criteria as suggested by the Organization for Economic Co-operation and Development [50]) were recruited via advertisements. All participants signed a written informed consent form and were compensated for their participation. Inclusion criteria included having right-hand dominance as assessed through The Edinburgh Handedness Inventory [51] and scoring higher than 3 in the Mini-Cog Test [52]. We excluded subjects who had a personal history of neurological and psychiatric disorders, had any contraindications related to HD-tACS assessed through our NIBS safety questionnaire, and had received tDCS or tACS in the previous three months. Participants completed the following motor performance upper limb screening assessment tests: Box and Block Test (**BBT**) [53], Purdue Pegboard Test (**PPT**) [54], and Handgrip Strength (**HGS**) [55].

### 2.2. Experimental Design

**Figure 1:**
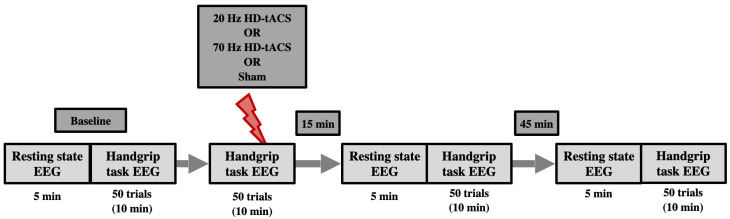
Schematic of the experimental timeline. Participants attended three different experimental sessions (each separated at least by a week), two active HD-tACS sessions (20 Hz and 70 Hz), and one Sham (control) session. Sessions were counterbalanced across participants. Each session started with baseline EEG recording for 5 min at resting state followed by 50 trials of the handgrip task. After that, active or Sham HD-tACS stimulation was applied while performing another 50 trials of the handgrip task. Resting-state EEG and handgrip-task EEG were performed again 15 min and 45 min post-tACS/Sham.

**Figure 2:**
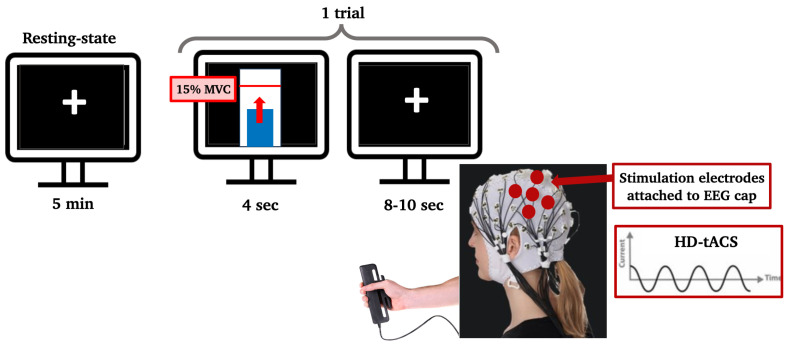
Computer screens showing the paradigm to the participant. During resting state, the participant looked at a black screen with a white cross in the center for 5 min. During the handgrip task, one trial consisted of reaching a threshold (red line inside the white bar), which was set to 15% of their maximum voluntary contraction, with a dynamometer using their right hand and staying on that threshold for 4 s. Each trial was followed by an inter-trial resting interval of 8 to 10 s. The stimulation electrodes that delivered HD-tACS were positioned on 5 recording electrodes over left M1 (anode: C3; cathodes: FC5, FC1, C3, CP5, and CP1).

The paradigm flow is shown in Figure 1. There were 3 experimental sessions in addition to the eligibility visit. A 64-channel EEG system (Brain Products, Gilching, Germany) was used to collect data, and stimulation was delivered using an EEG-compatible HD-tACS device (Soterix Medical, Woodbridge, NJ, USA). Baseline EEG at rest was recorded for 5 min, during which participants were seated in front of a screen displaying a centered white cross. They were asked to relax, look at the cross, and stay as still as possible. During the handgrip task, participants held a grip force response dynamometer with their right hand. They were required to squeeze a hand-clench dynamometer (BIOPAC, Goleta, CA, USA), which produced a linear force measurement output based on the pressure applied with the hand. A blue bar moved up and down according to the gripping force produced by the participants, who were asked to reach a red bar higher up as fast and accurately as possible. The force required to reach the target was 15% of their maximum voluntary contraction (**MVC**), which they had to hold for 4 s with an interval of 8–10 s resting between each handgrip (Figure 2). The handgrips were repeated 50 times (10 min). The hand dynamometer was connected to a BIOPAC system that converted the input to electrical signals. The signals were then transferred to a recording computer that displayed the force that was being applied by the participant in real time. Participants practiced until they understood the goal of the task (10 trials).

After the baseline recordings, participants received either 20 Hz, 70 Hz HD-tACS, or Sham stimulation for 10 min while repeating the 50 handgrip task as EEG signals were simultaneously recorded. The order in which participants received the type of stimulation was randomized. After the stimulation ended, participants were asked to fill out a questionnaire to monitor the following possible adverse effects of HD-tACS: headache, neck pain, scalp pain, tingling, itching, burning sensation, skin redness, sleepiness, trouble concentrating, and acute mood change [56]. Participants were also asked if they thought the stimulation was active or sham to assess for protocol blindness. EEG recordings (rest and handgrips) were repeated 15 and 45 min after stimulation ended.

### 2.3. Data Acquisition and Pre-Processing

EEG signals were amplified and sampled at 2500 Hz. All electrodes were referenced to FCz. Electrode impedances were kept below 20 kΩ. HD stimulation was delivered by a current regulator (Soterix Medical, Germany). The EEG cap covered the individual’s entire scalp, but the stimulation was delivered at pre-selected electrodes over the left sensorimotor cortex, with a target on M1 [28]. The anode was located in electrode C3 and the cathodes in FC5, FC1, CP5, and CP1. The stimulation lasted 10 min and it was delivered in the form of a sinusoid waveform with a peak-to-peak value of 1 mA and frequencies of 20 Hz and 70 Hz.

Offline EEG data were pre-processed using the Brainstorm MATLAB toolbox (version September 2024) [57]. Electrodes with an atypical power spectrum density were rejected from analysis. The rest of the EEG data were filtered (0.5–100 Hz bandpass, 60 Hz notch), resampled at 250 Hz, and then re-referenced to an average reference. Noisy segments (e.g., muscle, head, and jaw movement artifacts) were rejected by visual inspection. Independent components analysis (ICA) was used to identify and remove eye movement, muscle, and heart artifacts. Criteria for rejection included components’ topography and time history [58].

### 2.4. Data Analysis

#### 2.4.1. Behavioral Scores

Desrosiers et al. [59] developed predictive equations for PPT scores based on normative data resulting from their study. The normative data portion of the study involved 360 healthy participants over the age of 60 years. Student’s *t*-tests were used to determine if the predicted scores were significantly different from the scores obtained by our participants.

#### 2.4.2. Resting-State EEG

Signals from the resting state recording were epoched in 5 s segments (n = 51 ± 5.95/recording). EEG signals were convoluted using a Morlet wavelet transformation with a frequency range from 1 Hz to 55 Hz with 1 Hz steps (time resolution = 3 s; central frequency = 1 Hz) [60]. We further analyzed signals from electrodes FC3, FC1, C5, C3, C1, CP5, CP3, and CP1, since we were interested in the HD effects over M1 and surrounding areas. We excluded FC5, since it was a faulty electrode. The beta frequency was extracted (15–29 Hz) and averaged in this range to calculate the beta power at rest. We selected this range to avoid including any power from the contiguous alpha and gamma bands.

#### 2.4.3. Motor Task EEG

Signals were epoched from 1 s before to 8 s after the appearance of the blue bar that triggered the initiation of the handgrips. The first 5 trials were rejected for each subject and each recording. Additionally, trials were once again visually inspected and if still contaminated with artifacts they were manually rejected (n = 35 ± 6.18 trials/recording). EEG signals were then examined in the time–frequency domain using a Morlet wavelet transformation with a frequency range from 1 Hz to 55 Hz with 1 Hz steps (time resolution = 3 s; central frequency = 1 Hz) [60]. Time–frequency maps were averaged within the beta band (15–29 Hz). *MRBD* was calculated as follows:

(1) $$MRBD=\frac{P(t)-B}{B}\times 100\%$$

where *P*(*t*) is the absolute power at time *t* and *B* is the mean power of the baseline, which was defined as 0.9–0.1 s before the start of each trial. MRBD% was averaged over 0.5–3.5 s after the appearance of the visual cue during sustained contraction at 15% MVC [61]. The analyzed signals were focused on the same electrodes as the resting-state analysis. The EEG signals recorded during active HD-tACS were contaminated with very large artifacts; therefore, they were not included in the analysis.

### 2.5. Statistical Analysis

Repeated-measures analysis (**rmANOVA**) tests were conducted for beta features during rest and movement, with an a-level of 0.05 and factors Stimulation (20 Hz, 70 Hz, and Sham) and Time (baseline, post-15 min, and post-45 min). Post-hoc Bonferroni-corrected [62] *t*-tests were also used to test for differences across time (before applying stimulation vs. 15- and 45-min post-stimulation).

## 3. Results

### 3.1. Behavioral Assessment

The results from the behavioral assessments are reported in Table 1. The predicted scores for each of the participants based on their age were calculated according to Desrosiers et al. [59] (Table 2). There was no significant difference between the predicted scores of this model and the scores of our participants.

### 3.2. Participant Blinding

After the end of the stimulation, participants were asked if they believed they had received active stimulation and, if they believed they did, at which frequency. For all the sessions, 33% of participants correctly identified Sham stimulation, and 47% and 33% correctly identified the 20 Hz and 70 Hz active stimulation, respectively. Cochran’s Q test did not reveal significant differences between conditions (*p* = 0.716).

### 3.3. Effects of HD-tACS on Resting-State Beta Power

First, to test that our baseline measurements were comparable across stimulation conditions we ran Friedman tests (Table A1). The lack of significant differences supports the validity of our comparative analyses, as the starting points for each condition were not significantly different. The analysis of resting-state beta power across electrodes revealed varied effects of stimulation and time (Table 3. At electrode C3, there was a significant main effect of Stimulation (*F* = 0.087, *p* = 0.016), whereas no significant effects were observed for Time or the Time × Stimulation interaction. Similarly, electrode CP3 showed a significant main effect of Time (*F* = 3.958, *p* = 0.030) but no significant effects for Stimulation or their interaction. All other electrodes (FC3, FC1, C5, C1, CP5, and CP1) demonstrated no significant main effects or interactions (all *p* > 0.05).

**Figure 3:**
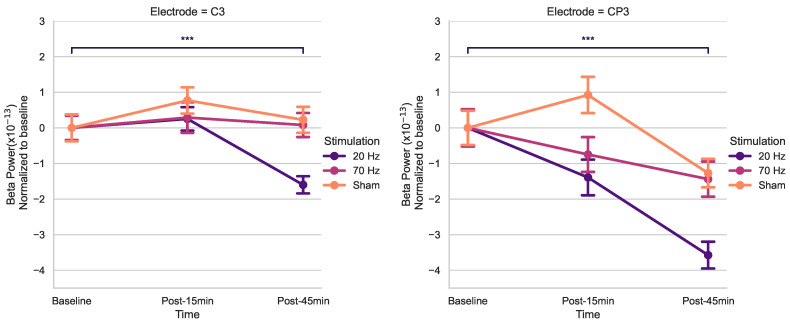
Effects of HD-tACS on average beta power at electrode C3 and CP3 normalized to baseline. Error bars represent standard error. Color indicates stimulation condition: orange = Sham, purple = 20 Hz, and pink = 70 Hz. (*** = *p* < 0.001).

Using post hoc *t*-tests of C3 and CP3 (Bonferroni-corrected) (Figure 3), spontaneous beta power significantly decreased only 45 min post-20 Hz HD-tACS (*p* < 0.001 in both electrodes) but not after 15 min (*p* > 0.05).

### 3.4. Effects of HD-tACS on MRBD

Baseline measurements were comparable across stimulation conditions as shown in Table A2. The lack of significant differences supports the validity of our comparative analyses, as the starting points for each condition were not significantly different. The results of the rmANOVA on MRBD across electrodes are summarized in Table 4. At electrode FC3, a significant interaction between Time and Stimulation was observed (*F* = 4.144, *p* = 0.005), while no main effects of Time or Stimulation were detected. Similarly, at electrode C3, a significant Time × Stimulation interaction was found (*F* = 2.694, *p* = 0.040), with no main effects. At all other electrodes, no significant main effects or interactions were observed (*p* > 0.05).

**Figure 4:**
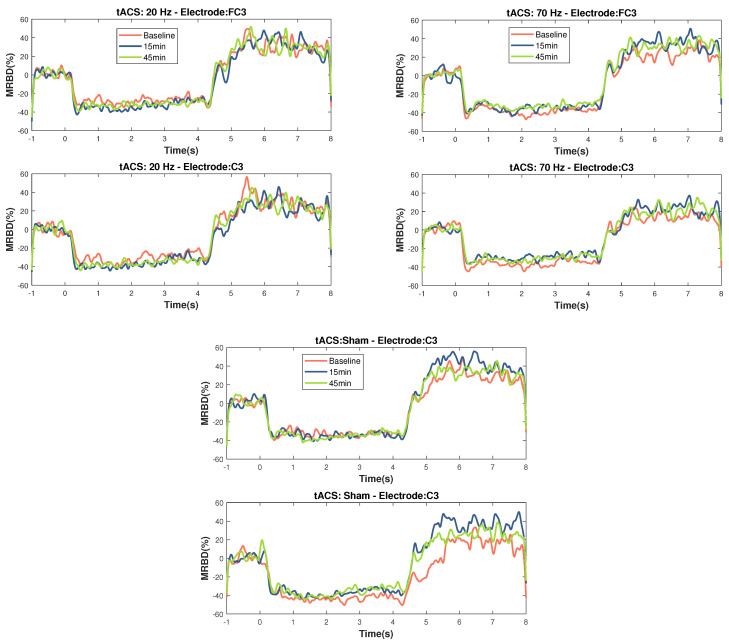
Temporal evolution of MRBD on electrodes that showed significant changes (C3 and FC3) across the different NIBS (20 Hz HD-tACS, 70 Hz HD-tACS, and Sham). Time zero is motor task onset and Time 4 is motor task offset. Applying 20 Hz HD-tACS induced a more negative MRBD only after 15 min and 70 Hz HD-tACS induced a more positive MRBD after 15 min in both electrodes and after 45 min only on FC3. No changes were significant during Sham stimulation.

The timecourses for MRBD at electrodes FC3 and C3 at the three time points during the three stimulation sessions can be seen in Figure 4.

**Figure 5:**
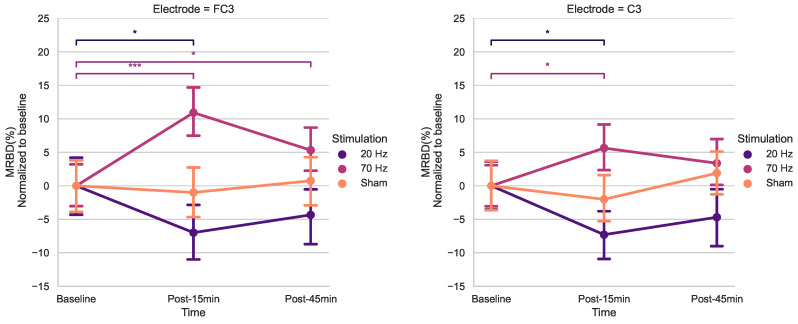
Effects of HD-tACS on average MRBD percentage at electrode FC3 and C3 normalized to baseline. Error bars represent standard error. Color indicates stimulation condition: orange = Sham, purple = 20 Hz, and pink = 70 Hz. (* = *p* < 0.05; *** = *p* < 0.001)).

Using post hoc t-tests for electrodes FC3 and C3 (Bonferroni-corrected) (Figure 5), a significant increase in MRBD percentage (more negative) was found 15 min post-20 Hz HD-tACS for both (FC3: *p* = 0.039; C3: *p* = 0.011). Applying 70 Hz HD-tACS elicited a decrease in MRBD percentage for both electrodes (FC3: *p* < 0.001; C3: *p* = 0.039), which persisted post-45 min only in FC3 (*p* = 0.036). There were no significant differences in MRBD values during Sham stimulation for either of them (*p* > 0.05).

## 4. Discussion

This study aimed to quantitatively examine the after-effects of HD-tACS on electrophysiological features in healthy older adults. Our rmANOVA anaysis for beta power revealed a noteworthy main effect of *Stimulation* for C3, as well as a significant effect of *Time* for CP3. Conversely, no other electrodes displayed any statistically significant main effects. Upon post hoc analysis of t-tests, a notable reduction in resting-state beta power was observed post-45 min for 20 Hz HD-tACS for both electrodes. In contrast, in the case of 70 Hz HD-tACS and Sham, no significant changes were observed in either electrode.

When analyzing MRBD effects, there was a significant *Time* × *Stimulation* interaction for FC3 and C3. The results of the post hoc t-tests showed higher MRBD values (more negative) 15 min after 20 Hz HD-tACS in both electrodes. Applying 70 Hz HD-tACS resulted in significant reductions in MRBD values after 15 min in FC3 and C3 and after 45 min only in FC3. Notably, no significant changes were observed during Sham stimulation. In the subsequent subsections, the implications of these results on beta oscillatory patterns are discussed in greater detail.

### 4.1. Population Behavioral Scores and Baseline Features

To ensure the representativeness of our participants concerning motor performance, we employed the Desrosiers et al. [59] model to predict the PPT score for each participant based on their age when performing the motor tests during the eligibility session. The PPT quantifies fingertip dexterity and gross movement of the hand, fingers, and arm. The predicted and actual scores of the PPT were similar in all the subtests, which suggests that the motor performance of our participants fell within the range of normal scores for the older population.

### 4.2. Modulation of Resting-State Beta Power

After administering 20 Hz HD-tACS, a delayed reduction in beta power was observed for electrodes C3 and CP3. This modulation appeared only 45 min post-stimulation, with no change detected at 15 min, suggesting that the impact on beta power is gradual rather than immediate. This finding aligns with previous studies showing no after-effects on beta power within 5 to 20 min after 20 Hz tACS [63]. However, prior research demonstrated that tACS could have effects on endogenous EEG power in the range of the stimulation frequency up to 70 min after the stimulation [64], which guided our decision to monitor changes up to 45 min post-stimulation.

The delayed response may reflect plasticity mechanisms, long-lasting modifications in neural connections and activity, with dynamic brain changes emerging over time. These results are consistent with reports of late plasticity changes in corticospinal excitability following 20 Hz tACS [65]. A relationship between beta oscillations and corticospinal excitability has been observed, with increased spontaneous beta oscillatory activity linked to smaller motor-evoked potential (**MEP**) amplitudes [66]. Furthermore, beta tACS has been shown to elevate cortical excitability in the M1 during stimulation, as evidenced by increased MEP amplitudes [67,68]. Heise et al. [41] also reported that more focal HD stimulation is significantly more effective in modulating MEPs post-stimulation. Additionally, delayed effects induced by tACS may be attributed to spike-timing-dependent plasticity (**STDP**), a process where the precise timing of neural activity determines whether synaptic connections are strengthened or weakened [69]. According to STDP principles, synapses in circuits resonating at frequencies similar to repetitive inputs are strengthened during stimulation. After stimulation ends, these synaptic modifications persist, leading to enhanced neural activity at the circuits’ resonant frequencies. This aligns with findings that beta tACS can sustain elevated beta oscillations and cortical excitability for at least an hour post-stimulation [64,65,70]. Currently, evidence on STDP and its specific manifestations in aging populations is limited. However, studies have shown that synaptic plasticity mechanisms, including STDP, may be less efficient in older adults, potentially due to age-related changes in synaptic connectivity, neurotransmitter levels, and cortical excitability [71]. This could influence the extent and timing of tACS-induced plasticity effects. While our findings do not directly investigate these mechanisms, they provide a basis for future research to explore how aging might modulate tACS effects through STDP-related processes.

Our results disagree with recent research showing that 20 Hz HD-tACS increased beta power following stimulation of the visual [72] and parietal cortexes [73]. Also, a study reported no significant effects when applying tACS at 20 Hz, also in M1 during rest, on the beta power in younger participants using the standard double-electrode tACS montage [74]. We can identify three possible reasons why our results differ from the aforementioned studies. First, the difference in age of our participants is a possible reason, as it has been shown before that older groups demonstrated a decrease in tACS-induced neuroplasticity compared to a younger cohort [75]. We chose an older population, since these HD-tACS frequencies have not been studied before in aging, and we specifically examined its effects on beta oscillations. Secondly, in our study, HD-tACS was applied while participants were performing a handgrip task (online) as opposed to HD-tACS during rest (offline). Differences have been observed across studies comparing tACS-induced changes in online and offline protocols [46]. Additionally, a recent study comparing online and offline HD-tDCS showed that only the online stimulation reduced the power of the alpha rhythm during motor skill execution [76]. Thirdly, we used a different electrode montage and it has been shown that an HD-tACS electrode montage delivers a more focal current to M1 than the standard double-electrode tACS montage [77,78]. It is important to note that beta-tACS has been shown to have mixed results on other outcomes, such as corticospinal excitability and motor function [79]. Therefore, more research should focus on applying HD-tACS at this frequency, while keeping similar parameters to the ones used in studies showing significant effects.

For 70 Hz, there were no significant changes in resting beta power, which does not align with our initial hypothesis. This null finding underscores a critical limitation in current HD-tACS research: the application of fixed-frequency stimulation across diverse neural populations. Sugata et al. [32] found frequency-specific increases in beta power, while our results are more consistent with Mastakouri et al. [80], who demonstrated that gamma-tACS effects are heterogeneous and subject-specific. The recent literature increasingly emphasizes the importance of individualized stimulation protocols. Multiple studies [69,81,82,83,84] have shown that tACS delivered at a subject-specific frequency can produce more robust effects on cortical oscillations compared to fixed-frequency approaches. Moreover, age-related neuroplasticity differences may further complicate our findings. Guerra et al. [85] demonstrated that gamma stimulation mechanisms differ between younger and older adults, with older participants showing enhanced motor skill consolidation despite potentially reduced long-term potentiation (LTP)-like plasticity.

One promising solution emerges in the form of closed-loop brain–computer interfaces (BCIs). As highlighted by Xu et al., these systems provide a dynamic approach to neural modulation by continuously monitoring outcomes and adaptively modifying stimulation parameters [86]. By identifying specific neural biomarkers in real time, such as oscillatory patterns in gamma or beta frequency ranges, closed-loop systems can adjust stimulation accordingly. This approach addresses the current limitations of fixed-frequency stimulation by enabling real-time adaptation to an individual’s unique neural dynamics. These nuanced findings suggest that future research should achieve the following:

- Develop individualized stimulation protocols based on baseline neural characteristics;
- Consider age-related neuroplasticity differences;
- Employ a more comprehensive assessment of stimulation effects;
- Explore closed-loop systems for real-time neural modulation.

Our null findings for 70 Hz stimulation should not be interpreted as a complete absence of effect, but rather as an invitation to develop more sophisticated, personalized neuromodulation approaches [87].

### 4.3. Modulation of MRBD

We found that after 15 min of 20 Hz HD-tACS, MRBD values became more negative in FC3 and C3. Conversely, after 15 min of 70 Hz HD-tACS, MRBD values became more positive in both FC3 and C3, and the effect persisted after 45 min only in FC3. These results align closely with our initial hypothesis. To the best of our knowledge, there are no other studies investigating the specific effects of HD-tACS on MRBD values in older adults. A recent study demonstrated that tACS at 10 Hz enhanced MRBD during a motor imagery task compared to pseudo-stimulation, indicating the capability of tACS to modify movement-related brain oscillations [88]. Additionally, other forms of NIBS, such as tDCS, have been shown to induce more negative MRBDs during motor imagery after 15 min of stimulation.

Xifra-Porxas et al. [13] previously mentioned that the motor performance decline observed in healthy aging may not be due to an impairment in the capacity to modulate beta oscillations. In fact, they observed a larger modulation in older compared to younger adults. On the other hand, beta oscillations at rest are greater in older adults [17], which suggests that increased desynchronization is needed to reach a threshold to initiate a movement. This would mean that modulating this desynchronization could later translate into a change in motor performance. While our study primarily focused on brain oscillations as a potential mechanism of HD-tACS-induced effects, we also evaluated motor task performance through measures of accuracy and reaction time. However, no significant changes were observed, potentially due to the simplicity of the motor task employed. Future research aiming to validate this hypothesis should utilize more complex motor tasks, such as the sequential visual isometric pinch task (**SVIPT**), which better resembles the level of difficulty encountered in everyday life skills [89]. Tasks like the SVIPT have been shown to challenge motor learning and performance to a degree comparable to real-world activities [90], making them ideal for studying the interplay between MRBD, beta modulation, and motor performance.

Finally, the fact that electrode C3, the anode of our HD-tACS montage, showed significant changes in both resting-state beta power and MRBD values suggests a focalization of the current right on the electrode that delivers the current. This focalization possibly results from a reduced distribution of the electrical field compared to the conventional tACS montage, which utilizes two distant patch electrodes [41].

Taken together, the results of this study are partly aligned with our initial hypothesis. In terms of beta power, only 20 Hz HD-tACS showed a decrease in beta power, but only 45 min after the end of stimulation, while 70 Hz HD-tACS did not show any significant changes, contrary to our hypothesis that it would decrease beta power. Regarding MRBD, both 20 Hz and 70 Hz showed the expected results: the former resulted in a more negative MRBD and the latter in a more positive. However, these changes in beta oscillations may not necessarily translate to improvements in motor performance. We do not report effects on motor tasks, since the complexity of our task is not enough to show impacts. Future research should focus on assessing the impact of HD-tACS on motor performance using tasks that are more complex and reflective of real-world daily activities, particularly for older adults.

There are certain limitations in our study that may explain some of variability in our HD-tACS outcomes and previously reported outcomes, such as interindividual differences, including skull thickness and the actual amount of current that reaches the cortex [91,92]. Because of these differences, individualized stimulation frequencies and current amplitudes, validated by studies such as Yamaguchi et al. [93], emphasize the parameter-dependent nature of tACS effects. In addition, our limited stimulation duration (10 min) contrasts with longer-lasting effects seen in extended gamma-tACS in mice, suggesting prolonged sessions or multiple-day approaches akin to tDCS studies for in-depth exploration [49,94]. Finally, the analysis of ongoing brain signals during concurrent stimulation is of primary interest and would also shed light on the immediate effects and mechanisms of HD-tACS. Recent advances in artifact removal algorithms will enable this type of analysis in our data [95].

## 5. Conclusions

To our knowledge, this is the first HD-tACS study that looks at the beta oscillation effects on healthy older adults. Future research should focus on replicating protocols that have been shown to have an effect on the desired outcomes in a bigger cohort to establish a more robust effect. In summary, our study reveals that HD-tACS has a modulating effect on beta oscillations during movement. Notably, different HD-tACS frequencies led to specific alterations in MRBD values, indicating frequency-specific effects on movement-related brain oscillations. The focal impact observed at electrode C3, the site of HD-tACS anodal stimulation, underscores the technique’s precision over brain regions related to motor control. Future studies should explore personalized protocols tailored to individual neural characteristics, potentially employing advanced tACS methods such as HD-tACS, phase-shifted tACS, amplitude-modulated tACS, temporally interfering, and intersectional short-pulse techniques. Addressing these intricacies will enhance our understanding of tACS efficiency, guiding its optimized application in clinical settings. This research not only contributes to the ongoing discourse on brain stimulation but also holds promise for therapeutic interventions, making HD-tACS a promising avenue for exploration in diverse clinical populations such as stroke and PD patients. Further investigations in these domains will unveil the full potential of HD-tACS as a targeted therapeutic tool. Exploring HD-tACS effects in healthy older adults is crucial in providing insights into designing targeted interventions, addressing the complex interplay between neural oscillations, brain stimulation, and motor performance in the aging population.

## Figures and Tables

**Table 1:** Subject characteristics and behavioral scores.

	Mean ± SD	Min–Mix
Age (years)	69.7±4.2	65–78
Handedness (/100)	94.6±6.8	80–100
MiniCog (points)	4.7±0.5	4–5
BBT Right Hand (blocks)	56.4±5.5	46–65
BBT Left Hand (blocks)	57.2±6.9	43–65
PPT Right Hand (pins)	13.4±2.3	9–17
PPT Left Hand (pins)	12.0±2.4	6–15
PPT Both Hands (pins)	10.4±2.0	6–14
PPT Assembly (pins)	26.7±5.1	17–34
HGS Right Hand (kg)	32.7±11.3	17.6–55.6
HGS Left Hand (kg)	30.7±9.5	15.6–54.3

BBT = Box and Blocks Test, PPT = Purdue Pegboard Test, HGS = Handgrip Strength.

**Table 2:** Participants’ PPT scores (predicted from Desrosiers’ [59] model vs. real scores).

PPT Subtests	Predicted	Real	*p*-Value	Cohen’s d
Right Hand	12.8±0.8	13.4±2.3	0.136	0.326
Left Hand	12.0±0.7	12.0±2.4	0.439	0.048
Both Hands	9.8±0.6	10.4±2.0	0.128	0.358
Assembly	26.7±2.2	26.7±5.1	0.493	0.005

PPT = Purdue Pegboard Test.

**Table 3:** Summary of rmANOVA results for resting-state beta power at each electrode.

Electrode	Main Effect of Time (F, *p*)	Main Effect of Stimulation (F, *p*)	Time × Stimulation Interaction (F, *p*)
FC3	0.751, 0.480	0.303, 0.740	0.207, 0.933
FC1	0.800, 0.458	1.654, 0.209	0.492, 0.741
C5	0.258, 0.773	0.666, 0.521	1.535, 0.204
C3	0.795, 0.461	0.087, 0.016	0.429, 0.786
C1	0.229, 0.796	0.112, 0.893	1.458, 0.227
CP5	0.751, 0.481	0.465, 0.632	1.221, 0.312
CP3	3.958, 0.030	0.137, 0.872	0.849, 0.500
CP1	2.206, 0.128	0.371, 0.693	0.586, 0.673

**Table 4:** Results of rmANOVA on MRBD at each electrode.

Electrode	Main Effect of Time (*F*, *p*)	Main Effect of Stimulation (*F*, *p*)	Time × Stimulation Interaction (*F*, *p*)
FC3	0.035, 0.965	1.276, 0.294	4.144, 0.005
FC1	0.577, 0.567	1.049, 0.363	2.367, 0.063
C5	0.492, 0.616	0.866, 0.431	1.896, 0.123
C3	0.616, 0.546	0.964, 0.393	2.694, 0.040
C1	0.079, 0.923	1.759, 0.190	1.271, 0.292
CP5	0.189, 0.828	0.827, 0.447	1.561, 0.197
CP3	1.449, 0.251	1.877, 0.171	2.156, 0.085
CP1	0.238, 0.789	0.340, 0.714	0.686, 0.604

## Data Availability

The raw data supporting the conclusions of this article will be made available by the authors on request due to ethical reasons, since we did not mention to our Ethics Board that we would make the data available. The code is publicly available in the following repository: https://github.com/kenyamelissamf/tACS_Aging (accessed on 20 November 2024).

## Abbreviations

The following abbreviations are used in this manuscript:

BBT	Box and Block Test
CMC	Corticomuscular Coherence
ECR	Extensor Carpi Radialis
EEG	Electroencephalography
EMG	Electromyography
FDI	First Dorsal Interosseous
GABA	Gamma-aminobutyric Acid
HD-tACS	High-Definition Transcranial Alternating Current Stimulation
HGS	Handgrip Strength Test
iAPF	Individual Alpha Peak Frequency
ICA	Independent Component Analysis
ISP	Intersectional Short Pulse
LTP	Long-term Potentiation
M1	Primary Motor Cortex
MEG	Magnetoencephalography
MEP	Motor-Evoked Potential
MPS	Mild Parkinsonian Sign
MRBD	Movement-Related Beta Desynchronization
MVC	Maximum Voluntary Contraction
NREM	Non-rapid Eye Movement
NIBS	Non-Invasive Brain Stimulation
PD	Parkinson’s Disease
PPT	Purdue Pegboard Test
rmANOVA	Repeated-Measures Analysis of Variances
SICI	Short-Interval Intracortical Inhibition
SVIPT	Sequential Visual Isometric Pinch Task
tACS	Transcranial Alternating Current Stimulation
tDCS	Transcranial Direct Current Stimulation
TI	Temporally Interfering
TMS	Transcranial Magnetic Stimulation

## Appendix A

**Table A1:** Baseline comparison of beta power across stimulation conditions.

Electrode	Chi-Square	*p*-Value	Median Beta Power ($\times 10^{-13}$)		
			**Sham**	**20 Hz**	**70 Hz**
FC3	0.400	0.819	1.103	1.235	1.248
FC1	0.933	0.627	0.670	0.803	0.849
C5	2.133	0.344	0.946	1.146	0.804
C3	0.133	0.936	0.699	0.761	0.702
C1	1.733	0.420	0.939	1.095	1.067
CP5	0.533	0.766	0.620	0.716	0.715
CP3	0.400	0.819	1.110	1.500	1.049
CP1	0.133	0.936	0.717	0.783	0.794

Note: Friedman tests showed no significant differences between conditions at baseline (all *p*-values > 0.344).

**Table A2:** Baseline comparison of MRBD across stimulation conditions.

Electrode	Chi-Square	*p*-Value	Median MRBD (%)		
			**Sham**	**20 Hz**	**70 Hz**
FC3	4.933	0.085	−21.459	−7.981	−25.445
FC1	4.133	0.127	−18.151	−7.596	−23.386
C5	3.733	0.155	−10.502	2.976	−12.650
C3	4.933	0.085	−20.433	−5.745	−21.876
C1	5.733	0.057	−28.285	−21.597	−33.539
CP5	2.800	0.247	−20.020	−5.455	−20.274
CP3	5.200	0.074	−24.733	−18.127	−28.332
CP1	0.933	0.627	−19.511	−11.305	−22.868

Note: Friedman tests showed no significant differences between conditions at baseline (all *p*-values indicated above).
